# Hematopoietic reconstitution of neonatal immunocompetent mice to study conditions with a perinatal window of susceptibility

**DOI:** 10.1038/s41598-018-30767-1

**Published:** 2018-08-16

**Authors:** Karen Laky, Philip Dugan, Pamela A. Frischmeyer-Guerrerio

**Affiliations:** Laboratory of Allergic Diseases, National Institute of Allergy and Infectious Diseases, National Institutes of Health, Bethesda, MD USA

## Abstract

Efficient hematopoietic reconstitution of wild type mice requires preconditioning. Established experimental protocols exist to transplant hematopoietic stem cells into lethally irradiated or chemically myeloablated adult mice or unirradiated immunodeficient mice. We sought to develop a protocol to reconstitute immuno-replete neonatal mice. We describe irradiation and injection procedures for two-day old mice that lead to efficient long-term reconstitution of primary and secondary lymphoid organs. We demonstrate that the frequencies of lymphoid and myeloid cells in primary and secondary lymphoid organs are indistinguishable from unirradiated uninjected sex- and age-matched control animals by 5 weeks post-reconstitution. Thus, this system will facilitate studies aimed at understanding the developmental and environmental mechanisms that contribute to conditions that have a window of susceptibility during the perinatal period.

## Introduction

In recent years, the importance of early life events on the subsequent development of human disease that may not manifest until late childhood or even adulthood has become increasingly appreciated^1^. The perinatal period is a critical developmental window where environmental exposures, epigenetic changes, microbial exposures, and infectious contacts can influence the development and function of multiple organ systems and thus susceptibility to diseases including obesity, chronic kidney disease, neuro-psychiatric conditions, cardiovascular disease, and allergic disease. Identifying the key pathogenic factors, determining their relative importance, and understanding their interplay is challenging. Animal models have several advantages over human subjects or *in vitro* models when studying complex disease states. Genetic and microbiota variations can be eliminated, dietary compliance can be ensured, and access to cells and tissues other than peripheral blood or cell lines allows a more comprehensive understanding of the condition and an opportunity to investigate tissue-specific phenotypes.

In many cases, interactions between immune cells and nonhematopoietic cells are essential for maintaining overall tissue homeostasis, and disruption of this communication may have critical importance in the pathogenesis of disease. For example, it is posited that infants become sensitized to food allergens, rather than develop tolerance, in part because of the immaturity of their gut tissues and altered interactions between intestinal epithelial cells and the immune system. The gastrointestinal tract of newborn mice closely resembles that of pre-term human babies and thus is often used in studies of gastrointestinal function^2,3^. Generation of bone marrow chimeras allows mixing and matching of recipient and bone marrow donors so that the role of hematopoietic and non-hematopoietic components can be studied alone or in combination, and in mice with a fixed genetic background without the 3-year delay incurred by completing 20 backcrosses^4^. The interactions between hematopoietic and non-hematopoietic cells often shape the nature and magnitude of immune responses, which can have long-term consequences on overall health.

We sought to establish an experimental protocol that allows complete hematopoietic reconstitution of newborn immunocompetent mice so that the relative contribution of hematopoietic and nonhematopoietic lineages could be evaluated during the perinatal period using any wild type or genetically modified strain as the donor or host. Hematopoietic cells can be efficiently transplanted into unirradiated immunodeficient fetal or neonatal mice^5–8^. Complete reconstitution of wild type mice requires pre-conditioning, regardless of the age of the recipient^8–10^ or the donor^11,12^. Numerous conditioning regimens have been tried, alone or in combination, with varying degrees of success including irradiation, cytokines^13^, antibodies^14–18^, DNA synthesis inhibitors^13^, and alkylating agents^19^. Taking into account previous data on mortality, frequency of mice with detectable donor cells, and the frequency of donor cells among total CD45^+^, as well as practical considerations such as time, expense, special training, and side effects, we chose to optimize an irradiation regimen.

## Results

During murine embryonic development, fetal liver is the primary source of hematopoietic progenitors. For a brief time during the perinatal period the liver continues to be a source of extra-medullary hematopoiesis until eventually the bone marrow is fully established and takes over^20^. We reasoned that donor bone marrow injected into the liver of lethally irradiated newborn mice should be able to seed primary lymphoid organs and eventually populate the peripheral immune system of wild type mice.

A harem breeder cage was established with 3 dams and 1 sire. Harem breeder cages were used rather than 1:1 breeder set-ups to eliminate differences in microbiota^21^. Dams were pulled from the harem cage when they appeared pregnant (~E12–14) and a new dam was added to the harem cage. Individually housed pregnant females were checked daily for new births. The first morning that pups were noted was designated Day 0. If a single sex of recipients is desired, male and female pups of all strains can be distinguished based upon anogenital distance^22^ or via PCR using DNA isolated from tail snips^23^. Alternatively, in pigmented strains, a dark spot is visible in the perineum area of male pups, but absent in females^24^.

Hematopoietic reconstitution of wild type mice requires preconditioning. In the absence of irradiation long-term engraftment of donor cells in wild type recipients occurs only rarely and is usually incomplete^8,10,25^. Lethal irradiation of adult mice is achieved with 8.5 G^26^. There are reports in the literature of neonates exposed to ≤600 rads surviving without reconstitution^11,27^. To determine the optimal dose of lethal irradiation for neonates, 2 day old wild type 129SvE pups (CD45.2) were exposed to increasing doses of gamma-irradiation, ranging from 300 to 1000 rads, using a cesium-137 source irradiator. To provide warmth and to minimize external odors from being transferred to pups during transport or irradiation, pups were placed in an irradiator pie along with a soiled nestlet and bedding material from their original cage. Two day old neonatal mice tolerated irradiation well. At 8 weeks of age, ≤15% mortality was observed for any of the doses of irradiation evaluated (Table 1). One litter of mice exposed to 900 rads and reconstituted on day 2 was allowed to age; 5 of 5 survived past 20 weeks.

**Table 1:** Survival and reconstitution rates.

Age of treatment	Dose rads	Survived to weaning	Reconstituted (≥10% donor BM at 8 wk post reconstitution)
Neonate	0	7/7	N/A
	0	9/9	0/9
	300	6/6	6/6
	400	5/5	2/5
	500	5/5	5/5
	750	9/9	7/9
	900	19/20	14/14
	1000	10/10	5/6
adult	900	16/16	16/16
	1000	13/13	13/13

Two day old (neonate) or adult (>5 weeks) wild type 129SvE mice (CD45.2) were exposed to increasing doses of gamma-irradiation ranging from 0 to 1000 rads. Following irradiation at the age and dose indicated, mice were reconstituted with CD45.1^+^ congenic total bone marrow cells.

Immediately following irradiation, pups were reconstituted with bone marrow cells harvested from CD45.1 congenic mice. Use of a congenic marker allows donor cells to be distinguished from residual host cells. The appropriate choice of congenic marker depends on the cell lineage(s) of interest. Expression variants of leukocyte common antigen (LCA, Ly5, CD45), CD45.1 and CD45.2, allow most leukocyte cell lineages to be distinguished by flow cytometry. Other strategies commonly employed to evaluate chimerism include ubiquitous markers such as sex chromosomes^28,29^, glucose phosphate isomerase or phosphoglycerate kinase isozymes^8,10,19,30,31^; Thy1.1 and Thy1.2 for T cells^32^; allelic variants of immunoglobulin for B cells^18^, or hemoglobin for erythrocytes^11,31,33^.

To eliminate the variable of cell dose, a saturating dose of bone marrow was injected. Seminal studies from the Weissman laboratory established that 1 × 10^6^ total bone marrow cells is sufficient to rescue ≥95% of lethally irradiated adult mice^26^. The maximum volume that could be accommodated by the liver of a 2-day old mouse was 30 μl. Bone marrow cells were harvested from the femurs of adult CD45.1 congenic mice. On average, 5 ± 1 × 10^7^ live bone marrow cells were recovered from each donor mouse. Cells were resuspended at ≥3 × 10^7^ cells/ml in sterile 1X phosphate buffered saline (PBS) so that 30 μl of inoculum would contain ≥6 × 10^6^ BM cells, which was >5-times excess of saturation. The inoculum was filtered through sterile 50 μM nylon mesh. To allow efficient loading of the syringe and account for dead space within the syringe and the needle, ≥50 μl bone marrow cells were prepared for each intended recipient.

To perform the intra-hepatic (i.h.) injections, 3 pups at a time were sedated by cold. A square of sterile gauze was placed on top of wet ice. Pups were placed on top of the gauze until gross movement ceased, which took approximately 5 minutes. Each pup was lightly scruffed on the back in the shoulder area and held in a supine position to visualize the abdomen. The stomach of nursing pups appears as a large milky white spot on the right side of the abdomen. To the left and slightly above the stomach, the liver appears as a large reddish organ just below the rib cage. Using a 0.3 cc syringe with a 29 gauge ½″ needle, 1 × 10^6^ bone marrow cells resuspended in 30 μl of sterile 1X PBS were injected i.h. The needle was inserted bevel up to allow visualization of the inoculum being injected. After the proper volume was dispensed, to prevent backflow the needle was held in place for a few seconds before being withdrawn slowly. Sterile gauze was used to apply gentle pressure to the injection site for several seconds. Pups were wrapped in nestlet material from their original cage, placed on top of a warming pad until movement resumed, and then were returned to their mother where they remained until weaning at 21 days of age. If a dam was not immediately attentive to the returning pups, the pups were marked with the dam’s scent by encouraging her to urinate on to the pups or on to the investigator’s gloved hands which were then used to rub the pups’ skin.

**Figure 1:**
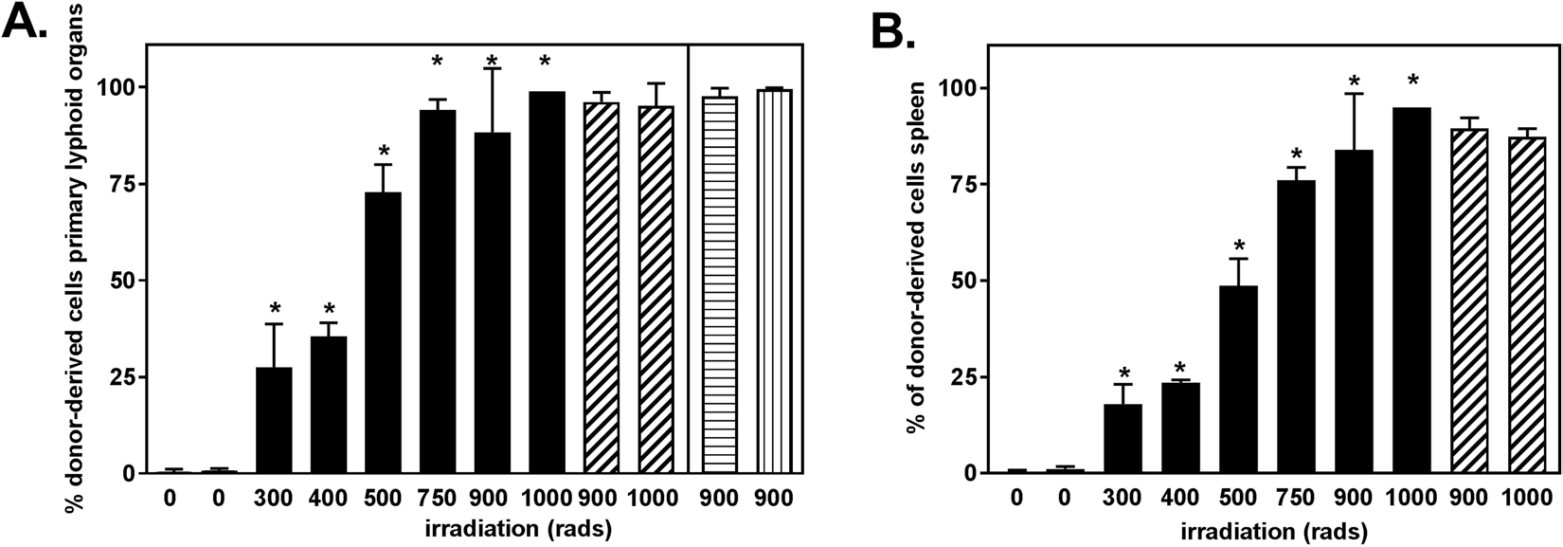
Donor cell reconstitution is positively correlated with the dose of irradiation. Wild type 129SvE mice (CD45.2) were exposed to increasing doses of gamma-irradiation, from 0 to 1000 rads. Following irradiation at the dose indicated, pups were intra-hepatically (i.h.) injected with CD45.1^+^ congenic bone marrow cells. (**A**) The frequency of CD45.1^+^ donor-derived cells in primary lymphoid organs of reconstituted mice was determined 5–8 weeks post irradiation and reconstitution. White, black, and diagonally striped bars to the left of the line are bone marrow. White bar is bone marrow of unirradiated and uninjected control mice. Black bars are bone marrow of pups irradiated and injected at 2 days of age. Diagonally striped bars are bone marrow from mice irradiated and injected with congenic bone marrow as adults. Bars to the right of the line are thymus from mice irradiated and injected with bone marrow at 2 days of age (horizontal lines) or as adults (vertical lines). Data shown are the mean ± SD of 2–10 mice analyzed individually. *p < 0.05, Dunnett’s multiple comparisons test. (**B**) The frequency of CD45.1^+^ donor-derived splenocytes in reconstituted mice was determined 5–8 weeks post irradiation and reconstitution. White bar is spleen from age matched unirradiated and uninjected control mice. Black bars are pups irradiated and injected at 2 days of age. Striped bars are spleen from mice irradiated and injected with bone marrow as adults. Data shown are the mean ± SD of 2–10 mice analyzed individually. *p < 0.05, Dunnett’s multiple comparisons test.

Mice were analyzed to determine the extent of hematopoietic reconstitution (Table 1). Studies have traditionally defined engraftment as >1% donor cells in peripheral blood^14,34^. We set a more rigorous standard and defined reconstitution as >10% donor-derived cells in the bone marrow at 5–8 weeks post-reconstitution, a timepoint when bone marrow and peripheral cells have achieved steady state^35–37^. No donor-derived cells were detected in animals that received i.h. injection in the absence of irradiation. Donor-derived cells were observed in all mice irradiated prior to i.h. injection. The extent of donor cell reconstitution in bone marrow and spleen were positively correlated with the dose of irradiation (Fig. 1A,B).

**Figure 2:**
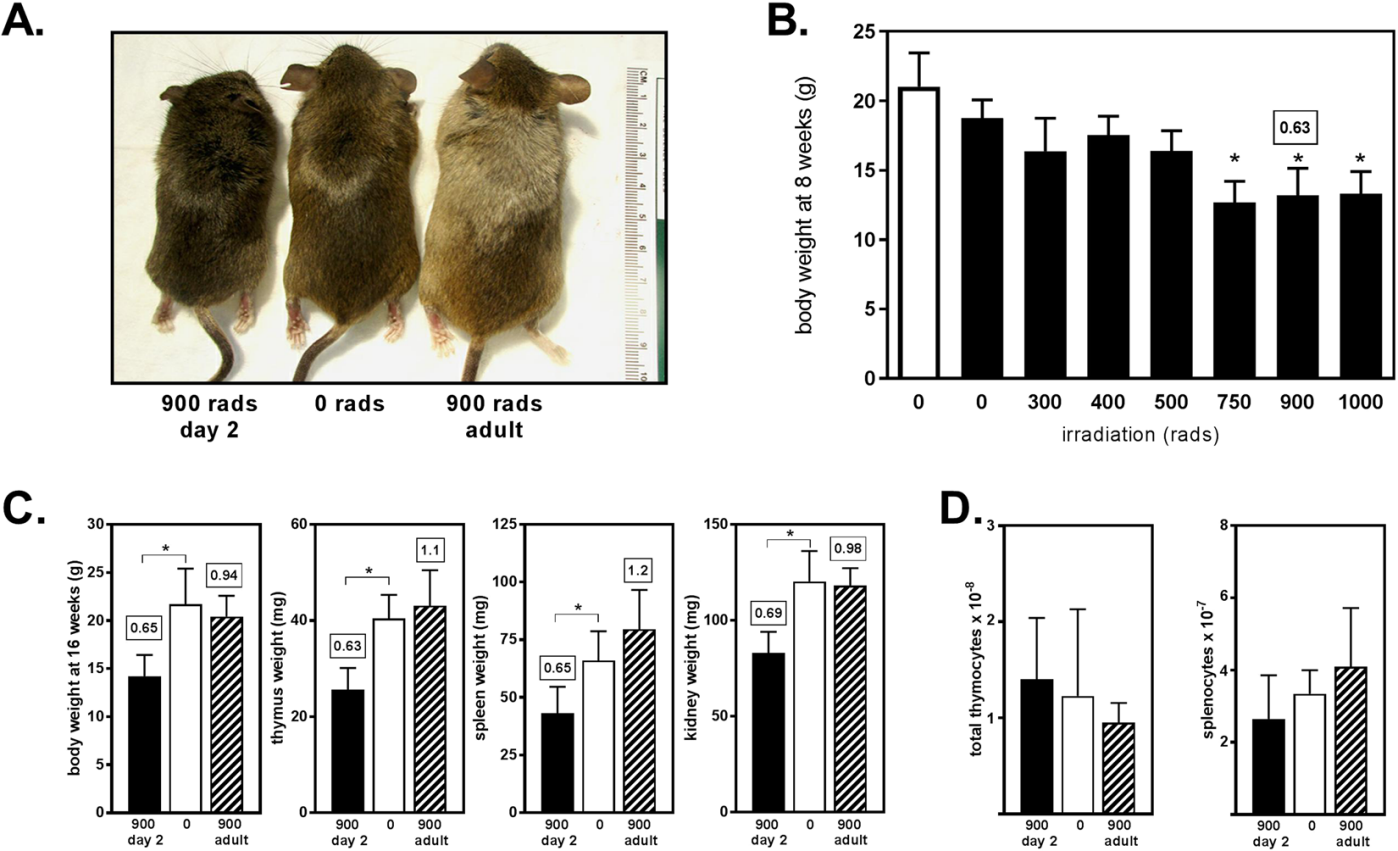
Total body irradiation impairs growth. (**A**) 8 week old mice that had been neonatally irradiated with 900 rads (900 rads day 2) were smaller than unirradiated adult mice (0 rads) and irradiated adult mice (900 rads adult), but appeared overall healthy and maintained an agouti coat color. (**B**) The total body weight of 5–8 week old mice that had received ≥500 rads on day was significantly less than non-irradiated, age- and sex-matched control animals. White bar is age-matched unirradiated and uninjected control mice. Solid bars are pups irradiated and injected at 2 days of age. Data shown are the mean ± SD of >4 mice per group, analyzed individually. Text box above the bar for 900 rads on day 2 = (mean experimental/ mean unmanipulated controls). *p < 0.05, Dunnett’s multiple comparisons test. (**C**) Primary and secondary lymphoid tissues and non-hematopoietic organs were proportionately decreased in size of 16–20 week old mice that had received 900 rads on day 2 (black bar) as compared to non-irradiated, age- and sex-matched control animals (white bar). In contrast, there were no significant differences between mice irradiated with 900 rads and reconstituted as adults (diagonally striped bar) and non-irradiated control animals (white bar). Data shown are the mean ± SD of >4 mice per group, analyzed individually. Numbers in boxes above the bars = (mean experimental/mean unmanipulated controls). *p < 0.05, Dunnett’s multiple comparisons test. (**D**) The number of total live leukocytes isolated from spleen or thymus did not differ significantly between irradiated and reconstituted mice and unmanipulated controls. Data shown are the mean ± SD of N ≥ 9 mice per group, analyzed individually 8–9 weeks post-reconstitution or 12–16 weeks of age.

Proliferating cells are most susceptible to the effects of irradiation and during the first week of life extensive proliferation occurs in multiple organ systems. Neonatal exposure to high dose irradiation impairs growth which manifests as a decrease in linear height and an overall decrease in body size^34,38–40^. Mice receiving higher doses of irradiation appeared smaller than controls (Fig. 2A). We weighed mice 5–8 wk after irradiation and observed that the overall size of the mice varied inversely with the dose of irradiation (Fig. 2B). Consistent with previous studies characterizing the effects of early life exposure to irradiation on growth^38,40^, at 8 weeks of age mice that had been exposed to ≥500 rads as a neonate were significantly smaller than non-irradiated, age- and sex-matched control animals (p < 0.01).

Despite being small, neonatally irradiated and reconstituted mice appeared overall healthy, walked with a normal gait, had normal dentition, and maintained an agouti coat color (Fig. 2A). The mass of primary and secondary lymphoid tissues and non-hematopoietic organs were proportionately decreased (Fig. 2C and^38^). In addition to weighing the organs, we made single cell suspensions. The number of total live leukocytes in spleen or thymus did not differ significantly between mice irradiated and reconstituted on day 2 and unmanipulated controls (p = 0.14 and p = 0.63, respectively) (Fig. 2D). The thymus of mice irradiated and reconstituted at 8 weeks of age and then analyzed 8 weeks post-reconstitution at 16 weeks of age had slightly fewer thymocytes and slightly more splenocytes, but the differences did not reach statistical significance (p = 0.24 and p = 0.26, respectively).

**Figure 3:**
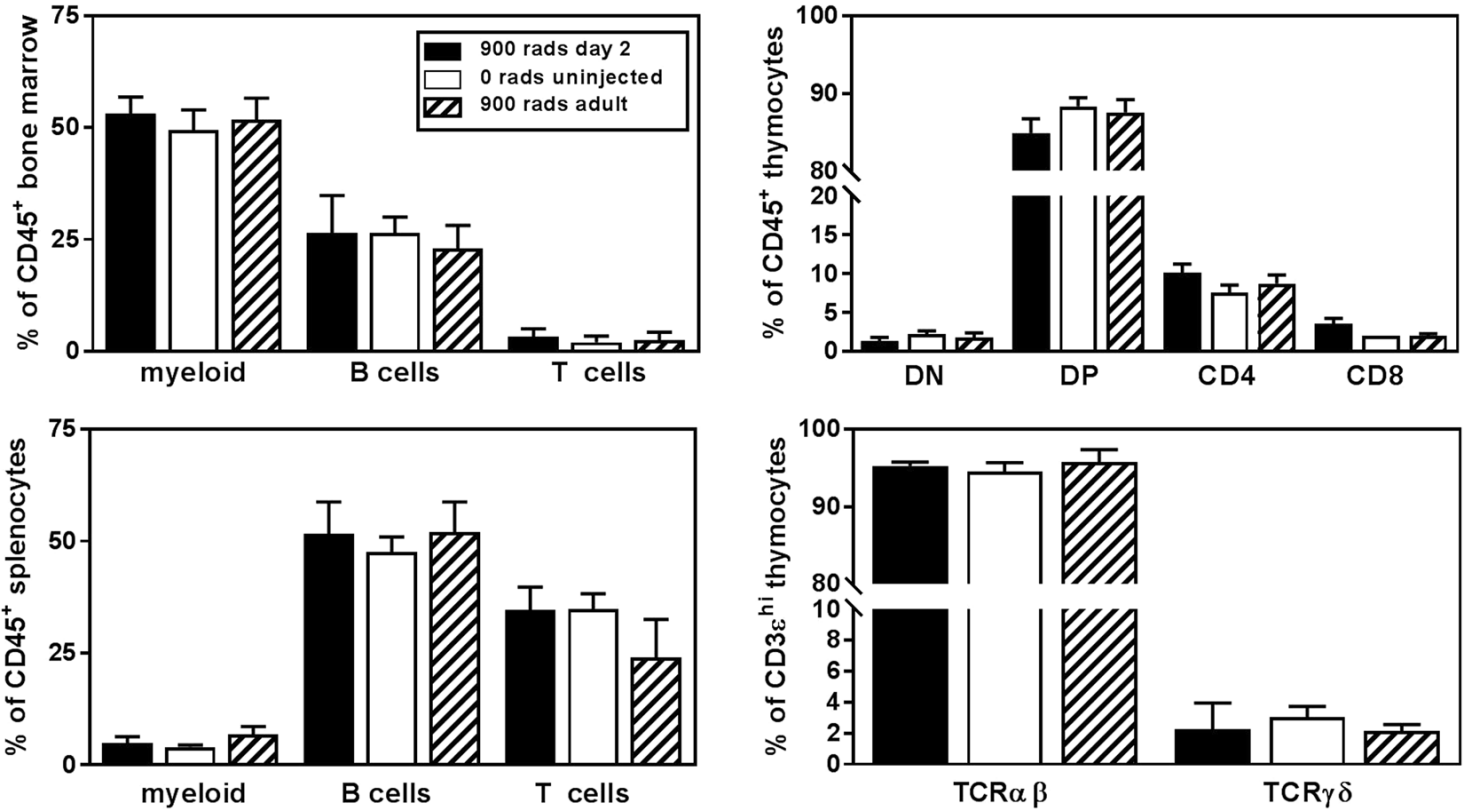
In 8 week old mice that had been neonatally irradiated with 900 rads (black bars), the relative contributions of donor derived B cell (CD45.1^+^ CD19^+^) and myeloid lineage cells (CD45.1^+^ CD11b^+^) developing in the bone marrow were indistinguishable from unmanipulated controls (white bars) or donor derived cells in adult irradiated and reconstituted mice (hatched bars); the relative composition of donor derived mature T (CD45.1^+^ CD3ε^+^), B (CD45.1^+^ CD19^+^), and myeloid (CD45.1^+^ CD11b^+^) splenocytes was the same. The frequency of donor derived T cell subsets in the thymus was unperturbed in 8 week old mice that had been neonatally irradiated with 900rads; double negative, DN (CD45.1^+^ CD4^−^CD8α^−^); double positive, DP (CD45.1^+^ CD4^+^CD8α^+^); CD4 single positive, CD4SP (CD45.1^+^ TCRβ^hi^ CD4^+^ CD8α^−^); CD8 single positive, CD8 SP (CD45.1^+^ TCRβ^hi^ CD4^-^CD8α^+^); TCRαβ (CD45.1^+^ CD3ε^hi^ TCRβ^+^); and TCRγδ (CD45.1^+^ CD3ε^hi^ TCRδ^+^). Data shown are the mean ± SD of ≥5 mice per group, analyzed individually.

We analyzed the cellular composition of donor-derived (CD45.1^+^) cells in the bone marrow, thymus, and spleen of mice that had been irradiated with 900 rads and reconstituted with bone marrow 8 weeks prior (Fig. 3). The frequency of lymphoid and myeloid lineage cells in bone marrow was indistinguishable from unirradiated age- and sex-matched control animals or adult irradiated and reconstituted mice. There were no significant differences in the frequency of immature (CD4^−^CD8^−^, CD4^+^CD8^+^) or mature T cell subsets (γδ, CD4^+^αβ^+^, CD8^+^αβ^+^) in the thymus of neonatally irradiated and reconstituted mice when compared to unirradiated controls or adult irradiated and reconstituted mice. The relative contributions of mature T cell, B cell, and myeloid lineage cells in the spleen were also indistinguishable from unmanipulated controls or adult irradiated and reconstituted mice.

Most animals exposed to total body irradiation have some residual host cells. Hematopoietic cells differ in radiosensitivity^41,42^. Most residual host cells in the lymphoid organs of lethally irradiated animals are radioresistant mature T cells (Supplementary Fig. 1A,B and^12,43–46^. In contrast, B cells, eosinophils, and CD4^+^CD8^+^ thymocytes are so radio-sensitive that exposure to even a sublethal dose of irradiation is sufficient to eliminate host-derived cells^42,47,48^. Several mechanisms may contribute to radio-resistance including cell cycle status at the time of irradiation, expression of anti-apoptotic genes, lineage/tissue specific tissue niches^46,49^.

## Discussion

In summary, we describe a protocol that consistently results in highly efficient hematopoietic reconstitution of lymphoid and myeloid lineages in immunocompetent neonatal mice. In contrast to antibody depleting regimens^16–18^ or chemotherapeutic regimens such as 5-fluorouracil or Busulfan^8,13^ the protocol is both cost and time efficient while consistently achieving nearly 100% survival with >75% donor engraftment. Compared to intrauterine injection protocols which involve survival surgery and result in high mortality rates^8,10,14,25^, the method that we describe requires minimal advanced training, has excellent survival rates, and does not require timed pregnant females.

Irradiation is commonly used for pre-transplant conditioning. However, total body irradiation is not without consequence^50^. Tissues with highly proliferative cells are most the most impacted. In neonates this includes long bones^40,51^. Exposure to total body irradiation is associated with decreased stature across species^34,38,39,52,53^. It is generally agreed that growth impairment correlates directly with the dose of irradiation and inversely with the age of exposure^40,54^. The mechanism(s) by which high dose irradiation stunts growth are not completely understood and are likely multifactorial. For instance, it has been posited that irradiation impairs growth hormone secretion. However, there is poor correlation between growth hormone levels and growth impairment in long term survivors^52,53^. Irradiation can damage epiphyseal growth plates which compromises bone growth including vertebrae, ribs, and leg bones^55–58^. To control for the pleiotropic effects of irradiation, it is essential to include the appropriate experimental controls, such as irradiated neonates reconstituted with syngeneic bone marrow.

The protocol was optimized by using unfractionated adult bone marrow as a source of progenitors because donors were readily available, and the number of donor cells was not a limiting factor which allowed a range of irradiation doses to be evaluated. Fetal liver or purified stem cells could also be used as a source of hematopoietic precursors. Data obtained from studies designed to directly compare the repopulation potential of different precursors have concluded that the repopulation potential of 500 hematopoietic stem cells (Lineage^neg^ Thy1^lo^Sca1^+^) or ~1.5 × 10^4^ total embryonic day 14.5 fetal liver cells is comparable to 1 × 10^6^ total adult bone marrow cells^12,26,59^. Detailed protocols for isolating hematopoietic stem cells from fetal or adult tissues and the expected frequency of precursors from different tissues at different ages are reviewed in^60^. The system is also amenable to making mixed chimeras, which are a powerful tool to study regulatory relationships and/or competitive fitness.

This system allows us to identify, manipulate, and evaluate hematopoietic and non-hematopoietic elements that are hypothesized to influence the development of multiple disease states, at developmentally relevant time points, and in an experimental system that takes into account the variables of genetics, microbiota, and diet. It alleviates the need to use immuno-deficient recipients, while still allowing the study of events occurring during the perinatal period. Cutaneous exposure to food antigen during the neonatal period is correlated with an increased risk of allergic sensitization^61–63^. Colonization of the gut occurs in the neonatal period, and the intestinal microbiome may influence susceptibility to food allergy^64–66^. The model we described will be a powerful tool to evaluate the role of microbiota and Toll-like receptors (TLRs) in allergic sensitization. TLRs, which are expressed on both hematopoietic and non-hematopoietic cells, are pattern recognition receptors that sense bacterial products. Neonatal mice reconstituted with TLR4-deficient bone will help to determine whether the increase in TLR4^+^ cells in the intestinal lamina propria of children with food allergy has physiological relevance^67^. TLR4-deficient mice reconstituted with WT bone marrow can be used to determine how TLR4 expression on keratinocytes or intestinal epithelial cells influences food allergy^68,69^. TLR9 agonists may have both prophylactic and therapeutic value for the treatment of peanut allergy^70–72^. Reconstituting TLR9 deficient neonates^73^ with WT bone marrow, or WT neonates with TLR9-deficient bone marrow, should reveal whether TLR9 expression on hematopoietic cells and/or non-hematopoietic cells (such as epithelial cells) plays a role in allergy versus tolerance to food antigens^74,75^. Understanding the cellular mechanisms involved in food allergy versus oral tolerance will help to optimize therapies for food allergy.

The focus of our studies is food allergy, but this model is well suited for studying any condition or organ system in which critical developmental changes occur during the postnatal period. For example, early life exposures are linked to airway disease later in life, and like the GI tract, the respiratory tract also undergoes critical changes during the post-natal period^76,77^. Brain structures like the cerebellum and hippocampus develop postnatally in mice and humans^78^, and thus the model system described here could be used to test hypotheses that postnatal inflammation and responses to infection impact the development of neuro-psychiatric conditions in adulthood such as autism, schizophrenia, depression, anxiety, obesity, and sleep disorders^79^. Clinical data show that there are sex-associated differences in disease course and prognosis of newborns^80^. Reconstituting female neonates with bone marrow from male donors, and the converse, would help distinguish between environmental (hormonal effects) and immune cell intrinsic effects conferred by genes located on the sex chromosomes including TLRs, several cytokine receptors, and Foxp3^81^. Thus, this methodology may have significant utility in efforts to unravel the complexity of the perinatal factors that influence long-term health.

## Methods

### Mice

129SvE (CD45.2) mice were obtained from the NIAID breeding contract at Taconic Farms and then bred in our animal facility. Mice were maintained under specific-pathogen-free conditions. All experiments were performed in compliance with the protocols and regulations of NIAID’s Animal Care and Use Committee who approved this study (Animal Study Protocol #LAD-11E).

### Flow Cytometry

Thymus and spleens were harvested in Media 199 supplemented with 10% fetal calf serum, 0.2 mM L-glutamine, 100 units/mL of penicillin, 100 µg/mL of streptomycin, and 250 ng/mL of Amphotericin B. Bone marrow cells were flushed from femurs. Single-cell suspensions were made by passing cells through 100 μM nylon mesh.

For surface staining, cells were resuspended in Hank’s Balanced Salt Solution supplemented with 0.1% bovine serum albumin +0.1% NaN_3_ and properly diluted antibodies. Live cells were gated based on exclusion of 7-Aminoactinomycin D. Data were collected on a LSR Fortessa cell analyzer and then analyzed in FlowJo (Treestar).

Antibodies used were: anti-mouse CD16/32 (2.4G2), CD45.1 (A20), CD45.2 (104), CD11b (M1/70.15), CD3ε (17A2), CD19 (1D3), TCRβ (H57-597), TCRγδ (GL3), CD4 (RMA4-5), and CD8α (53-6.7).

### Statistics

Statistical significance between groups was determined using unpaired two-tailed Student’s *t*-tests or Dunnett’s multiple comparison tests in GraphPad, PRISM. Probability values <0.05 were considered statistically significant.

### Ethical approval

The authors declare that all experimental protocols were approved by the National Institute of Allergy and Infectious Diseases’ Animal Care and Use Committee and were carried out in accordance with all rules and regulations stipulated by the National Institutes of Health Office of Laboratory Animal Welfare.

## Electronic supplementary material

Supplementary Figure
