# Supplementary material for: Hematopoietic reconstitution of neonatal immunocompetent mice to study conditions with a perinatal window of susceptibility
Source: Sci Rep. 2018 Aug 16;8:12254. doi: 10.1038/s41598-018-30767-1 (PMC6095844; doi:10.1038/s41598-018-30767-1)
Supplement: Supplementary file 1 — Supplementary Figure [file 41598_2018_30767_MOESM1_ESM.pdf]

Hematopoietic reconstitution of neonatal immunocompetent mice to study conditions with a perinatal window of susceptibility.

Karen Laky[1], Philip Dugan[1], and Pamela A. Frischmeyer-Guerrerio[1].
[1]Laboratory of Allergic Diseases, National Institute of Allergy and Infectious Diseases, National Institutes of Health, Bethesda, MD.

Supplemental Figure 1.
Most residual host cells in spleen or thymus are mature T cells.
Donor bone marrow-derived (CD45.1+) or residual host (CD45.2+) cells isolated from an 8 week old mouse that had been neonatally irradiated with 900rads and reconstituted with bone marrow on day 2.
(A) In contrast to splenocytes from unmanipulated controls or donor-bone marrow, most residual host cells in spleen were (CD3ε+) mature T cells.
(B) In contrast to unmanipulated controls or donor bone marrow-derived thymocytes, the majority of residual host (CD45.2+) cells in thymus were mature CD4 single positive (CD4+CD8α-) T cells.

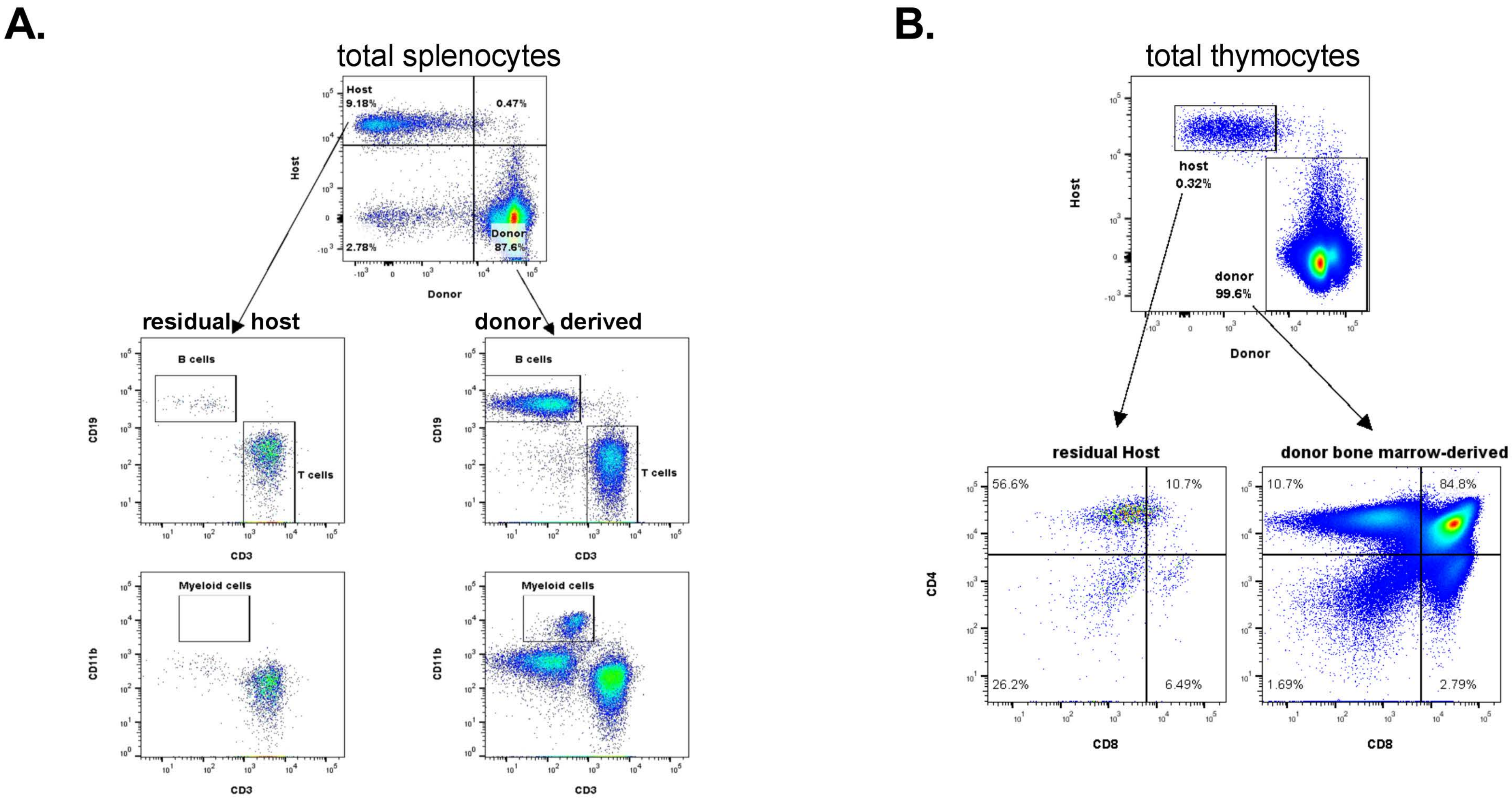
A.
total splenocytes
Host
9.18%
0.47%
2.78%
Donor
87.6%
Donor
residual host
donor derived
B cells
T cells
CD19
CD3
Myeloid cells
CD11b
CD3
B.
total thymocytes
host
0.32%
donor
99.6%
Host
Donor
residual Host
donor bone marrow-derived
56.6%
10.7%
26.2%
6.49%
10.7%
84.8%
1.69%
2.79%
CD4
CD8
